# Quality of couple relationship and associated factors in parents of NICU-cared infants during the first year after birth

**DOI:** 10.1038/s41372-024-02076-1

**Published:** 2024-08-03

**Authors:** Christine Persson, Jenny Ericson, Mats Eriksson, Raziye Salari, Renée Flacking

**Affiliations:** 1 School of Health and Welfare, Dalarna University, Falun, Sweden; 2 Centre for Clinical Research Dalarna, Uppsala University, Uppsala, Sweden; 3 Faculty of Medicine and Health, School of Health Sciences, Örebro University, Örebro, Sweden; 4 Department of Clinical Psychology and Psychotherapy, Friedrich-Alexander-Universität Erlangen-Nürnberg, Erlangen, Germany; 5 Child Health and Parenting (CHAP), Department of Public Health and Caring Sciences, Uppsala University, Uppsala, Sweden

**Keywords:** Paediatrics, Risk factors, Health policy

## Abstract

**Objective:**

To describe factors associated with quality of couple relationships among parents of infants cared for in neonatal intensive care units (NICUs) 1 year after birth and examine the trajectory of the relationship quality compared to parents from maternity units (MUs).

**Study design:**

Longitudinally comparative cohort design. Parents answered surveys during the first year after discharge about the couple relationship, social support, and depressive symptoms.

**Results:**

Better social support and a hospital stay of 7–14 days were positively associated with the couple relationship in NICU mothers, whereas not having slept together with the partner and infant during hospitalization were negatively associated. Depressive symptoms were negatively associated with the relationship among NICU fathers. There were no differences in trajectory of the relationship quality between NICU and MU parents.

**Conclusion:**

To strengthen couple relationships, it could be important to improve social support, facilitate space and time for support, and enable togetherness during hospitalization.

## Introduction

For most parents, becoming a parent brings joy, completeness in becoming a family, and increased tenderness [1, 2]. However, the transition to parenthood can be challenging [3] and stressful [4] and may have a negative impact on the couple relationship, e.g., reduced marital quality and satisfaction [5, 6], increased marital strain, decreased sexual contentment [1], and increased risk of depression [7]. Factors positively associated with relationship satisfaction are female sex, younger age, higher education, better income, and social support [8, 9].

Support from the partner during times of distress is linked to greater intimacy and trust [10], has a buffering effect on the relationship [8, 11], and enhances self-rated health in mothers [12]. Meier et al. [13] concluded that it is not only important how well partners support each other during times of stress but also that their engagement in parenthood is equal. Inequality in household tasks has also been associated with poorer health in both parents [14, 15].

Parents of neonatally cared infants often face distressing situations in a new and unfamiliar environment [16]. Experiencing symptoms of psychological trauma or depression is common for parents in neonatal intensive care units (NICUs) [17, 18]. Thus, NICU parents have increased risks of strain in couple relationships. However, few studies have investigated the couple relationship during and after NICU hospitalization. Some studies suggest that parents’ psychological distress and increased symptoms of depression related to NICU hospitalization may lead to long-term negative effects on the couple relationship [19, 20], and that the quality of the couple relationship decreases from NICU admission to discharge [21, 22]. Stefana et al. [23] suggest that the NICU experience might strengthen the couple relationship if the parents share their emotional states with each other. Results show preterm birth can influence the couple relationship positively and negatively [22–27]. In this sense the parents can be important sources of support for each other but also vital sources of increasing concern and stress [24].

Although knowledge about factors associated with the quality of the NICU parents couple relationship is important for improving neonatal care and families’ long-term well-being, few studies have explored this issue. Therefore, the aim of this study was to describe factors associated with NICU parents’ quality of the couple relationship 1 year after birth. Further, we aimed to describe the trajectory in the quality of the couple relationship the first year after birth compared to parents of term and healthy infants. We hypothesized that parents of preterm/ill infants experienced significantly more strain in the couple relationship compared to parents of term/healthy infants.

## Methods

### Design

This is the second paper from an ongoing longitudinal comparative cohort study (PANC—Parenthood After Neonatal Care) over 3 years [25]. An advisory group of parents whose infants had been cared for in NICUs was involved in developing the study aim, design, and recruitment strategies. They also tested different validated instruments and the questionnaires and helped to interpret the study’s findings. The parents had experiences from different NICUs in Sweden. The parents’ infants were born extremely preterm, very preterm, or preterm. Ethical approval was obtained from the Swedish Ethical Review Authority (dnr: 2019-04367).

### Setting

Families from six NICUs and four maternal units (MUs) in the middle and south of Sweden participated in the study. The NICUs varied in level of care from level II (infants born from gestation week (gw) 32 and infants having milder breathing problems) to level III (infants born from gw 22 and infants having severe breathing problems requiring a ventilator). The families participating in the study were recruited during the COVID-19 pandemic when different visiting restrictions were in place at NICUs and MUs. At all six NICUs, the parents had access to their infants 24 h a day without any restrictions (unless they had COVID-19 symptoms). In five of the NICUs, both parents could stay at the unit day and night, but relatives, friends, and siblings could not visit the unit as they could before the pandemic. At some MUs, there were restrictions for the fathers/partners, such as not staying at the unit with the mother and infant during certain months. All MUs had visiting restrictions for siblings, relatives, and friends. The provision of services from hospital social workers and follow-up care of the infants functioned as usual.

### Recruitment

All parents at the participating NICUs and MUs were informed about the study during their hospital stay. The information was available in four languages (Swedish, English, Arabic, and Somali) and comprised brochures, posters, and a study website containing written information and video clips. Both the birthing and non-birthing parent were invited to participate if they met the following criteria: at least 18 years of age, could speak one of the four languages, had been discharged from the unit to home (and not to another health care facility), their infant did not need palliative care, and social services were not involved in taking care of the infant. From March 2020 to March 2021, 1 month after discharge, the parents were sent information about the study, a link to the study website, the study consent form, and the first questionnaire. Parents willing to participate filled in the consent form and returned it with the first questionnaire in a prepaid envelope.

**Fig. 1:**
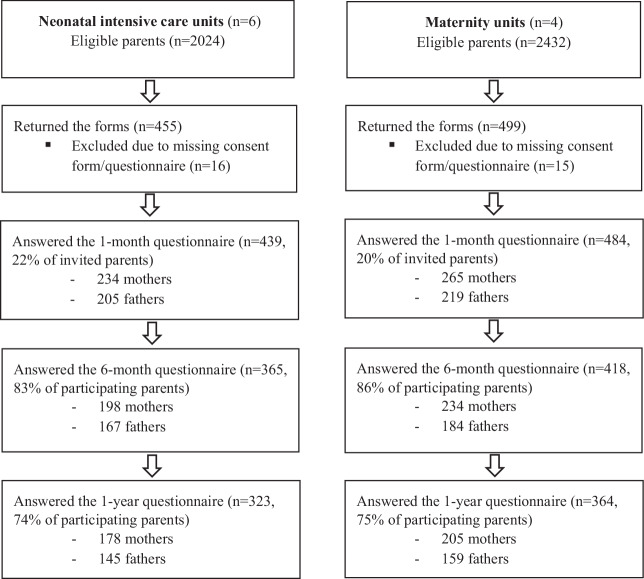
Flowchart of recruitment and number of participating parents. The left side shows eligible parents at the 6 NICUs and how many that answered the questionnaires at each timepoint. The right side shows eligible parents at the 4 MUs and how many that answered the questionnaires at each timepoint.

In total, 923 parents answered the first questionnaire 1 month after discharge and were included in the study: 439 parents from NICUs and 484 from MUs (22% and 20% response rates, respectively). The second questionnaire was administered 6 months after discharge and was answered by 783 parents (85%): 365 parents from NICUs and 418 from MUs. The third questionnaire was implemented when the infant was 1 year of age and was answered by 687 parents (74%): 323 parents from NICUs and 364 from MUs (Flowchart, Fig. 1).

In this project we chose not to use the term “partners” for fathers and non-birthing mothers as they are parents and not only partners. For simplicity, we hereafter refer to the birthing parents as mothers and non-birthing parents as fathers.

### Measures

The first questionnaire contained questions on parents’ sociodemographic background (gender age, education, occupation, civil status), experiences during pregnancy, birth, the NICU stay, and their health before and after birth. Questions were also asked about infant characteristics (length of hospital stay, gestational age, birth weight) and the infant’s health and care needs during the hospital stay and 1 month after discharge. The second and third questionnaires also included sociodemographic questions about civil status, occupation, health, household tasks, economy, and how long the parent had been home with the infant.

We used the Quality of the Dyadic Relationship (QDR36) [26] to assess the quality of the couple relationship. The QDR36 is a modified and validated version of the Dyadic Adjustment Scale (DAS) [27]. It contains 36 questions divided into five dimensions: consensus (11 items), cohesion (4 items), satisfaction (11 items), sensuality (5 items), and sexuality (5 items). Each item is scored between 1 (Never) and 6 (Always). Each dimension’s mean score ranges from 1.00 to 6.00 and the total index is the sum of the dimension’s mean scores, (5.00–30.00), with higher scores indicating better quality in the dyadic relationship. The QDR36 and DAS had been validated in Swedish and English contexts [26, 27]. The Edinburgh Postnatal Depression Scale (EPDS) [28] was used to assess depressive symptoms. The EPDS includes 10 questions about the participant’s past 7 days. Each question has four statements as answers. The statements are scored on a four-point scale (0–3) with a total sum ranging from 0 to 30, where higher scores indicate more depressive symptoms [28]. There are conflicting guidelines and usage of the cut-off values regarding purpose, population, sensitivity, and specificity [29–31]. We used the recommended cut-off values ≥10 for fathers [31] and ≥13 for mothers [32] in our analyses. The EPDS was available in the four study languages. All but the Somali version had been previously validated for women [29], and the English version had also been validated for men [31]. To assess parents’ social support, we used the Social Support Survey (MOS-SSS) [33]. The MOS-SSS consists of 19 items. Each item is scored on a five-point scale from 1 to 5, with answers ranging from “None of the time” to “All of the time”. The index is the total sum of all item scores, ranging between 19 and 95, divided into the number of items. The index total score can be between 1.00 and 5.00. The MOS-SSS was available and validated in two of the four study languages, English [33] and Arabic [34]. All the questions and instruments not available in the target language were translated forward to the target languages by professional translators, then translated back into the source language by other translators, inspired by the ISPOR procedure [35]. The original and translated versions were discussed with NICU staff and senior university lecturers who fluently spoke Swedish and the target languages until consensus was reached.

### Statistical analyses

The required sample size was calculated based on the hypothesis that NICU parents would be more likely to experience distress and problems in parental relationships 6 months after discharge, as indicated by lower scores on the QDR36 compared to parents of term and healthy infants. With alpha set at 0.025 (two-tailed) and power at 0.80, we needed to include parents of 393 infants per group (NICU and MU) to detect a small effect size (*d* = 0.2).

Descriptive statistics are given as frequencies and percentages. A missing completely at random (MCAR) test was performed to evaluate whether missing data were completely random for the outcome variable QDR36 and the variables chosen to be analyzed as predicted associated factors. The QDR36 showed the highest missing percentage (3.1%). The missing data were assumed to be random (*p* = 0.141); thus, no multiple imputation was performed [36]. The predicted factors chosen to be analyzed included NICU-specific factors and factors previously shown to influence the quality of couple relationships. A generalized linear model was used to analyze what factors were associated with the couple’s relationship (dependent variable). The results are presented with the change of mean at QDR36 (B), 95% confidence interval (95% CI) and *p* value for each factor.

A Linear mixed-effect model was used to analyze the trajectory of the QDR36 Index and its five dimensions at three time points (1 month, 6 months, 1 year) for four groups (NICU mothers, NICU fathers, MU mothers, MU fathers) and compare the groups (NICU mothers versus MU mothers and NICU fathers versus MU fathers). The model was adjusted for having older children living at home, multiple births, and having been treated for psychological symptoms during pregnancy. The results are presented with estimated marginal means and 95% CIs for each factor at each time point in the four groups, as well as *p* values for comparing the groups. Only participants who answered the 1-year questionnaire were included in the analyses. Missing cases were 9%. The level of significance was set at *p* ≤ 0.05 (two-tailed). IBM SPSS Statistics for Windows, Version 28.0 (IBM Corp, Armonk, NY, USA) was used in all analyses apart from the linear mixed-effect model analysis, where we employed R 4.3.0 (R Foundation for Statistical Computing, Vienna, Austria).

The datasets generated and analyzed during the current study are not publicly available due to an ongoing long-term project but are available from the corresponding author upon reasonable request.

## Results

### Characteristics of participating NICU parents

See Table 1 for detailed characteristics of the participating parents. The parents’ infants stayed in hospital between 1 and 150 days, and most of the infants (54%) were born at term (gw ≥37). Among parents of term infants, 96% of infants stayed in hospital for 1–14 days. Only 7–8% of the parents had very or extremely preterm infants (born between gw 23 + 0 and 31 + 6). All parents’ infants born between gw 23–27 had a longer hospital stay than 60 days, and among parents of infants born in gw 28–31, 89% of the infants had a longer hospital stay than 30 days. During the time in the NICUs, 63% of the mothers and 69% of the fathers reported staying at the unit for the whole or part of the time with their partner AND their infant.

**Table 1:** Characteristics of the participating parents who have attended NICU (*n* = 323) and MU (*n* = 364).

	NICU mothers (*n* = 178)		NICU fathers (*n* = 145)		MU mothers (*n* = 205)		MU fathers (*n* = 159)	
	*n*	%	*n*	%	*n*	%	*n*	%
Educational level								
Upper secondary school or less	65	36	76	52	71	35	91	57
Higher education	113	64	69	48	134	65	68	43
Born in Sweden								
No	14	8	14	10	19	9	13	8
Yes	164	92	131	90	186	91	146	92
Age								
21–28 years	44	25	18	12	51	25	23	14
29–34 years	98	55	71	49	103	50	76	48
35–42 years	36	20	56	39	51	25	60	38
First child								
No	83	47	65	45	116	57	87	55
Yes	95	53	80	55	89	43	72	45
Number of births								
Multiple	13	7	13	9	1	0.5	0	0
Singelton	165	93	132	91	204	99.5	159	100
Number of children in the household, including this child								
3–6 children	50	28	39	27	39	19	30	19
2 children	46	26	37	26	86	42	68	43
1 child	82	46	68	47	79	39	61	38
Duration as a couple when infant was born								
<5 years	63	36	51	35	85	42	67	42
≥5 years	114	64	94	65	120	58	92	58
Paying households expenses past 6 months								
Have had difficulties to pay	8	5	1	0.5	12	6	3	2
Have had no difficulties to pay	170	95	142	99.5	192	94	155	98
Months at home with children								
≤1 month	1	0.5	46	32	1	0.5	61	39
>1 month to ≤3 months	1	0.5	56	39	1	0.5	62	40
>3 months to ≤6 months	8	4.5	27	19	3	1.5	27	17
>6 months	168	94.5	16	11	197	97.5	6	4
Treated for mental health issues in the past 6 months								
Yes	26	15	9	6	21	10	5	3
No	152	85	136	96	183	90	154	97
EPDS ≥ 13 at 1 year								
Yes	10	6	6	4	16	8	9	6
No	167	94	135	96	187	92	148	94
EPDS ≥ 10 at 1 year								
Yes	30	17	18	13	36	18	18	11
No	147	83	123	87	167	82	139	89
Physical complications after birth at 1 year								
Yes	38	21	n/a	n/a	55	27	n/a	n/a
No	140	79	n/a	n/a	149	73	n/a	n/a
Gestational age at birth								
23–27 gw	4	2	3	2	0	0	0	0
28–31 gw	9	5	9	6	0	0	0	0
32–36 gw	69	39	55	38	4	2	4	3
>37 gw	96	54	78	54	201	98	155	97
Length of hospital stay after birth								
30–150 days	23	13	21	14	0	0	0	0
15–30 days	32	18	26	18	0	0	0	0
7–14 days	53	30	44	30	1	0.5	1	0.5
1–6 days (0–6 days for MU parents)	69	39	54	37	199	99.5	158	99.5
Slept together with partner and infant whole or part of the time at the hospital								
No	64	37	44	31	73	36	66	43
Yes	109	63	98	69	129	64	86	57
Child has an illness or impairment at 1 year								
Yes	10	6	8	6	1	0.5	3	2
No	167	94	136	94	202	99.5	155	98
Parent’s frequency of doing household duties								
Often or always	57	32	41	28	76	37	55	35
Sometimes or never	11	6	16	11	11	5	12	7
Half of the time	110	62	87	60	117	57	92	58
Social Support, MOS-SSS Index (*m*, SD)	4.27 ± 0.86		4.16 ± 0.82		4.25 ± 0.87		4.08 ± 0.85	

NICU parents were significantly more likely to be first-time parents (*p* = 0.009), have multiple births, a lower gestational week at birth, longer hospital stays, and have more infants with illness or impairment compared to MU parents (*p* < 0.001) (not presented in table).

### Factors associated with NICU parents’ quality of the couple relationship 1 year after birth

For mothers, the following factors were significantly associated with a more positive couple relationship (higher mean QDR36 index) 1 year after birth: being younger (*p* = 0.003), born outside Sweden (*p* = 0.02), having better social support (*p* = 0.01), and a hospital stay of 7–14 days at a NICU compared to 1–6 days (*p* = 0.04). Risk factors for having a lower mean QDR36 index included not sleeping together with the infant and father for the whole or part of the hospital stay (*p* = 0.04), and experiencing performing most of the household tasks (*p* = <0.001). For fathers, being younger was associated with a more positive dyadic relationship (*p* = 0.007). Risk factors for having a lower mean QDR36 index included an EPDS score of ≥10 (*p* = <0.001) and multiple births (*p* = 0.05) (Table 2).

**Table 2:** NICU parents Quality of Dyadic Relationship (QDR36) Index and associated factors when the infant is 1 year old, analyzed with a Generalized Linear Model (scale response linear).

	Mothers (*n* = 160)		Fathers (*n* = 123)	
	*B*, (95% CI)	*p* value	*B*, (95% CI)	*p* value
Educational level				
Upper secondary school or less	0.48 (−0.27 to 1.22)	0.21	−0.29 (−1.18 to 0.61)	0.53
Higher education	ref		ref	
Born in Sweden				
No	1.77 (0.28 to 3.25)	0.02	0.75 (−0.90 to 2.40)	0.37
Yes	ref		ref	
Age				
21–28 years	1.64 (0.56 to 2.72)	0.003	2.06 (0.57 to 3.55)	0.007
29–34 years	0.76 (−0.18 to 1.70)	0.11	1.09 (0.15 to 2.04)	0.023
35–42 years	ref		ref	
First child				
No	−0.25 (−1.04 to 0.53)	0.52	−0.20 (−1.18 to 0.77)	0.68
Yes	ref		ref	
Number of births				
Multiple	−0.62 (−2.06 to 0.82)	0.40	−1.49 (−3.00 to 0.15)	0.05
Singleton	ref		ref	
EPDS ≥ 13 at 1 year				
Yes	−0.98 (−2.54 to 0.58)	0.22	n/a	
No	ref		n/a	
EPDS ≥ 10 at 1 year				
Yes	n/a		−3.52 (−4.91 to −2.13)	<0.001
No	n/a		ref	
Length of hospital stay after birth				
30–150 days	0.91 (−0.27 to 2.09)	0.13	−0.92 (−2.34 to 0.51)	0.21
15–30 days	−0.35 (−1.36 to 0.66)	0.50	0.61 (−0.66 to 1.89)	0.34
7–14 days	0.91 (0.05 to 1.78)	0.04	0.68 (−0.44 to 1.81)	0.23
1–6 days	ref		ref	
Slept together with partner and infant whole or part of the time at the NICU				
No	−0.82 (−1.61 to −0.03)	0.04	−0.28 (−1.30 to 0.75)	0.60
Yes	ref		ref	
Parent’s frequency of doing household duties				
Often or always	−1.58 (−2.38 to −0.78)	<0.001	−0.57 (−1.56 to 0.42)	0.26
Sometimes or never	−1.30 (−2.87 to 0.27)	0.10	−1.28 (−2.72 to 0.16)	0.08
Half of the time	ref		ref	
Social Support, MOS-SSS Index (*m*, SD)	0.56 (0.11 to 1.01)	0.01	0.44 (−0.18 to 1.07)	0.16

Presented with estimated change of QDR36 (*B*), 95% confidence interval (95% CI) and *p* value for each variable.

### The trajectory of the couple relationship during the first year after birth

NICU parents’ QDR36 index slightly decreased from 1 month after hospital discharge until the infants were aged 1 year. The decrease in the QDR36 index was 0.8 for NICU mothers and 0.7 for NICU fathers (Table 3). For the comparison group of parents from MUs, the QDR36 index decreased by 0.9 points for both mothers and fathers. For all parents (NICU mothers and fathers, MU mothers and fathers), cohesion and sensuality decreased the most. Mothers rated sexuality significantly higher than fathers in both groups, and MU mothers rated consensus significantly lower than MU fathers. However, there were no significant differences between the NICU and MU parents in the trajectory of the quality of the couple relationship during the infants’ first year (Table 3). Thus, our hypothesis was not supported, as parents of preterm/ill infants did not experience significantly more strain in the couple relationship than parents of term/healthy infants.

**Table 3:** Estimated marginal means (EMM), 95% confidence interval (95% CI) and *p* values over Quality in Dyadic Relationship (QDR36) index and its five dimensions for mothers and fathers at NICUs and MUs using Linear mixed-effect models.

	NICU mothers *n* = 160	MU mothers *n* = 194	NICU mothers vs. MU mothers	NICU fathers *n* = 123	MU fathers *n* = 139	NICU fathers vs. MU fathers
	EMM (95% CI)	EMM (95% CI)	*p* value	EMM (95% CI)	EMM (95% CI)	*p* value
QDR36 Index, averaged over the level of time	**23.7 (23.4–24.1)**	**23.7 (23.4–24.1)**	**0.94**	**23.6 (23.2–23.9)**	**23.7 (23.3–24.0)**	**0.79**
1 month	24.2 (23.8–24.5)	24.2 (23.9–24.6)	0.74	23.9 (23.6–24.3)	24.1 (23.7–24.4)	0.61
6 months	23.6 (23.2–23.9)	23.6 (23.2–23.9)	1.00	23.5 (23.2–23.9)	23.6 (23.2–23.9)	0.85
1 year	23.4 (23.0–23.7)	23.3 (23.0–23.7)	0.86	23.2 (22.8–23.6)	23.2 (22.8–23.6)	0.99
Dyadic consensus, averaged over the level of time	**5.22 (5.16–5.28)**	**5.27 (5.21–5.33)**	**0.26**	**5.17 (5.11–5.24)**	**5.17 (5.10–5.23)**	**0.86**
1 month	5.25 (5.18–5.32)	5.32 (5.26–5.39)	0.12	5.20 (5.13–5.27)	5.21 (5.14–5.28)	0.76
6 months	5.20 (5.13–5.27)	5.25 (5.18–5.32)	0.32	5.16 (5.08–5.23)	5.14 (5.07–5.22)	0.84
1 year	5.21 (5.13–5.28)	5.23 (5.16–5.30)	0.66	5.16 (5.08–5.24)	5.12 (5.05–5.20)	0.47
Dyadic satisfaction, averaged over the level of time	**4.97 (4.90–5.04)**	**4.98 (4.91–5.05)**	**0.85**	**5.0 (4.93–5.08)**	**5.02 (4.96–5.09)**	**0.67**
1 month	5.05 (4.97–5.12)	5.09 (5.02–5.16)	0.46	5.05 (4.98–5.13)	5.10 (5.03–5.18)	0.34
6 months	4.96 (4.88–5.03)	4.95 (4.88–5.03)	0.96	5.01 (4.93–5.09)	5.02 (4.94–5.09)	0.86
1 year	4.88 (4.80–4.96)	4.87 (4.79–4.94)	0.78	4.93 (4.85–5.01)	4.93 (4.85–5.01)	0.96
Dyadic cohesion, averaged over the level of time	**4.88 (4.80–4.97)**	**4.90 (4.82–4.98)**	**0.77**	**4.89 (4.80–4.98)**	**4.91 (4.83–4.99)**	**0.72**
1 month	5.05 (4.96–5.15)	5.10 (5.01–5.19)	0.46	5.04 (4.95–5.14)	5.10 (5.00–5.19)	0.43
6 months	4.83 (4.73–4.93)	4.85 (4.76–4.95)	0.75	4.82 (4.72–4.92)	4.84 (4.75–4.94)	0.71
1 year	4.72 (4.62–4.82)	4.69 (4.60–4.79)	0.71	4.76 (4.66–4.87)	4.74 (4.64–4.85)	0.77
Dyadic sensuality, averaged over the level of time	**4.89 (4.79–4.99)**	**4.89 (4.79–4.98)**	**0.96**	**4.87 (4.77–4.97)**	**4.93 (4.83–5.03)**	**0.43**
1 month	5.05 (4.94–5.16)	5.03 (4.93–5.14)	0.81	5.03 (4.92–5.14)	5.07 (4.97–5.18)	0.58
6 months	4.82 (4.70–4.93)	4.85 (4.74–4.96)	0.68	4.80 (4.68–4.92)	4.90 (4.78–5.01)	0.25
1 year	4.76 (4.64–4.87)	4.73 (4.63–4.84)	0.76	4.73 (4.61–4.85)	4.77 (4.65–4.88)	0.65
Dyadic sexuality, averaged over the level of time	**3.75 (3.67–3.84)**	**3.76 (3.68–3.84)**	**0.89**	**3.61 (3.52–3.71)**	**3.67 (3.58–3.75)**	**0.40**
1 month	3.76 (3.66–3.86)	3.79 (3.70–3.88)	0.64	3.63 (3.53–3.73)	3.71 (3.61–3.81)	0.27
6 months	3.75 (3.65–3.85)	3.72 (3.62–3.81)	0.63	3.66 (3.55–3.77)	3.67 (3.57–3.78)	0.87
1 year	3.75 (3.64–3.86)	3.78 (3.68–3.88)	0.72	3.54 (3.43–3.66)	3.61 (3.51–3.72)	0.34

The bold values shows the QDR36 Index and its five dimensions averaged change over the level of time.

Measured 1 month and 6 months after discharge and when the infant was 1 year old. Adjusted for, having older children living at home, multiple births and treated for psychological symptoms during pregnancy.

## Discussion

This study is unique in that it focuses on couple relationships after having a preterm or ill infant discharged from a NICU, specifically, but also compared to parents of healthy and term infants. Our study shows no significant differences in the quality of the couple relationship up to 1 year after birth between parents of preterm/ill infants and parents of term/healthy infants. Both groups of parents rated the quality of their relationship as high, and their rating only decreased slightly during the first year of the infant’s life.

The present results are discussed in light of previous research and the Bowen Family Systems Theory (BFST) [37]. In the center of the theory is the individual and the nuclear family (one-generation parents and their children). Surrounding the nuclear family is the extended family (closest family members and friends) [37, 38]. Emotional processes govern relationships within couples, families, and the extended family. The theory emphasizes that the interplay between individuality and togetherness is important within relationships. It affects stability, cohesiveness, and cooperation. When the relationship is balanced, individuals put equal amount of life energy into the relationship, which increases togetherness [37]. Everyone in the system affects each other, and those in the nuclear family affect each other most [37, 38].

Our findings show that better social support from significant others (family and friends) was associated with a better couple relationship among mothers when the infant was 1 year of age. Previous studies have shown that family and friends are important sources of support for parents in the NICU [39]. Even so, there are often restrictions on who can visit, how many visitors, and how long they can stay [40, 41]. NICUs often lack guidelines or routines for how family and friends can be involved, despite the importance of the support family and friends can provide [42]. In line with the BFST the extended family can be *“a significant stabilizing force”* for the nuclear family ([37], p. 267), which highlights the importance of receiving support from significant others [37]. Hence, including family and friends in the NICU could be important in promoting better social support and strengthening the long-term relationship between couples.

A hospital stay between 7 and 14 days compared to 1 to 6 days was associated with a better couple relationship 1 year after birth in mothers. In a Swedish study, mothers whose infants had longer hospital stays showed significantly lower risks of postpartum depression symptoms 4 months after discharge [43]. According to the BFST, people manage reactions and anxiety primarily through relationships [37]. Hence, parents with longer hospital stays may have more time to process the birth of their infant together, receive support and counseling from staff, and support each other before coming home to other household duties and work.

Further, findings from the present study show that mothers who did not sleep together with their partner and infant during the whole or parts of their hospital stay, rated the couple relationship lower than the mothers who did sleep with their partner and infant. We do not know why some parents slept with their partner and infant and some did not. One explanation might be that they had other children at home that needed care. There is also a possibility that parents who already had a good relationship also had a higher preference for sleeping and staying together than parents who struggled in their relationship. However, sharing experiences and understanding what the partner goes through might increase intimacy, trust, and the opportunity to support each other. Kerr and Bowen state that the interplay between individuality and togetherness leads to emotionally significant relationships [37]. Thus, it must be seen important to begin the parental journey in togetherness to share the experience of becoming parents. If both parents could stay in the unit together, they have a better chance of investing equal energy into the relationship and giving each other emotional and practical support, previously shown to be associated with a better couple relationship [8, 11, 13, 37]. In Sweden, parents have good possibilities of staying together at NICUs because they both receive temporary parental leave during hospitalization. In our study, nearly 70% of NICU fathers stayed at the NICUs together with their partner AND infant for all or part of the hospitalization period.

In our study the number of parents with symptoms of depression was small, regardless of gender. Accordingly, the results must be interpreted cautiously. Fathers’ depression symptoms were associated with lower quality of their couple relationship, an association confirmed in previous studies [4, 7]. Also, according to the BFST, a family member’s physical or psychological symptoms affect the other family members. How they are affected depends on their individuality [37]. Screening both parents for symptoms of postnatal depression after discharge might lead to better-tailored support, which can be beneficial for individuals with mental health issues, as well as the couple relationship and the whole family in the long term.

### Strengths and limitations

This study has several strengths. In this study an advisory group of NICU parents was involved in developing the aim, design, recruitment strategies, testing questionnaires, and interpreting the results. Thus, the advisory group contributed with new and important perspectives, increasing the study’s relevance, credibility, validity, and findings. Over 920 parents consented to participate in the study and answered the first questionnaire. In addition, 85% answered the second questionnaire and 74% answered the third, which are acceptable follow-up rates. The demographics of the parents were similar in the NICU and MU groups. Parents who did not speak Swedish were also included because all information and the questionnaires were available in four languages. Our results, in which the quality of the couple relationship was measured at 6 months, correspond well with those of Hansson and Ahlborg [6], who also used the QDR36 but in a general population of first-time parents. The mean index scores in their and our study were between 23 and 24 points 6 months after delivery.

The study also has limitations. The participation rate in the first questionnaire was low, 20–22% of eligible parents consented to participate. A national survey for similar populations in Sweden has corresponding response rates (23%) despite being much shorter [44]. The low participation rate concerns the representativeness and, consequently, the generalizability of the results. Our study lacks data on nonparticipating parents. Therefore, we do not know the extent of bias in our sample. However, there is a high risk that participating parents in our study had a higher education level than the general population, which might benefit couple relationships [8]. Regardless, the education levels were similar between NICU parents and MU parents, so higher education should have influenced the results equally in both groups. Moreover, infants born extremely or very preterm correspond with the rates in Sweden in general [44]. The fact that the families were admitted to the hospital during the first year of the COVID-19 pandemic might have influenced their concerns and health negatively. Due to the MU’s visiting restrictions, the NICU parents had better options to be together during hospitalization. Parents had the possibility to stay at NICU together, both during the Covid-19 pandemic and in general, which can be seen as a limitation regarding the generalization to other countries with less beneficial social support systems.

## Conclusion

No differences were seen in the quality of couple relationships during the first year after discharge between parents of preterm/ill infants and parents of term/healthy infants. Several factors were associated with the long-term quality of couple relationships in parents of NICU-cared infants, including support from family and friends, hospitalization for 7 to 14 days, staying together at the NICU, and depressive symptoms. Hence, it must be seen important improving family and friends’ support, facilitating time and space for support, and enabling togetherness during hospitalization, because it may strengthen the couple-relationship long-term.

Long-term studies on families’ relationships, support needs, and parenting are warranted after discharge from NICU settings, where both parents can be together 24/7 with their infant.

## Data Availability

The datasets used and analyzed during the current study are available from the corresponding author upon reasonable request.
